# Data Mining of Prognostic Microenvironment-Related Genes in Clear Cell Renal Cell Carcinoma: A Study with TCGA Database

**DOI:** 10.1155/2019/8901649

**Published:** 2019-10-30

**Authors:** Bin Chen, Wei Chen, Jing Jin, Xueping Wang, Yifang Cao, Yi He

**Affiliations:** Urology Department, Jiaxing First Hospital, First Affiliated Hospital of Jiaxing University, Jiaxing, Zhenjiang 314001, China

## Abstract

Clear cell renal cell carcinoma (ccRCC) is one of the most prevalent kidney malignancies. The tumor microenvironment (TME) is highly related to the oncogenesis, progress, and prognosis of ccRCC. The aim of this study was to infer the level of infiltrating stromal and immune cells and assess the prognostic value of them. The gene expression profile was obtained from TCGA and used for calculating the stromal and immune scores. Based on a cut-off value, patients were divided into low- and high-stromal/immune score groups. Survival analysis was performed to evaluate the prognostic value of stromal and immune scores. Moreover, differentially expressed genes (DEGs) that are highly related to TME were determined and applied for functional enrichment analysis and protein-protein interaction (PPI) network. The Kaplan-Meier plot demonstrated that patients with high-immune scores and stromal scores had poorer clinical outcome. In addition, a total of 89 DEGs were identified and mainly involved in 5 pathways. The top 5 degree genes were extracted from the PPI network; among them, IL10 and XCR1 were highly associated with prognosis of ccRCC. The results of the present study demonstrated that ESTIMATE algorithm-based stromal and immune scores may be a credible indicator of cancer prognosis and IL10 along with XCR1 may be a potential key regulator for the TME of ccRCC.

## 1. Introduction

Renal cell carcinoma (RCC) is the most prevalent kidney malignant tumor globally [1], and it is estimated that over 350,000 cases are diagnosed with RCC each year [2]. Clear cell renal cell carcinoma (ccRCC) is the most common and invasive form in all RCC and comprises about 70–80% of all RCC cases [3, 4].

In recent years, the tumor microenvironment (TME) has already attracted a huge amount of interest from researchers. TME is a complicated system which consists of an extracellular matrix, stromal cells (like fibroblasts, occasionally adipocytes, and mesenchymal stromal cells), and immune cells (such as B and T lymphocytes, macrophages, and natural killer cells) [5]. Immune and stromal cells are the most important component of nontumor cells in TME and have shown a potential value for diagnosis and prognosis prediction of cancers [6, 7]. Previous studies have found that the extent of stromal cells could provide a prognostic factor for patients with solid cancers [8]. In addition, it also has been reported that activated CD8+ T cell density in TME is associated with favorable clinical outcomes of ccRCC [9, 10]. Nevertheless, several immune cells have the opposite effect. For example, the recruitment of CD4+ T cells in TME could promote RCC proliferation through modulating TGF*β*1/YBX1/HIF2*α* signals [11]. Moreover, regulatory T cells (Tregs) can also inhibit tumor immune responses by releasing immunosuppressive cytokines [12]. Macrophages have been reported to have crucial function in both promoting and blocking cancer growth. Macrophage M2 presenting CD163 and CD204 is highly associated with poor prognosis of RCC [13], whereas Hutterer et al. found that tumor-associated macrophages could independently reduce the risk of death in RCC [14].

Immunohistochemistry (IHC) and flow cytometry are the most commonly used technology for determining immune and stromal cells in TME by detecting marker proteins [15]. However, due to the restriction of the channel of markers, conventional technology could not evaluate diverse immune cells simultaneously [16]. As an alternative, algorithms based on a large scale of gene expression profile have been applied for predicting tumor purity in many researches [17]. The ESTIMATE (Estimation of Stromal and Immune cells in Malignant Tumor tissues using Expression data) algorithm developed by Yoshihara et al. is a new tool for inferring the level of infiltrating stromal and immune cells by calculating stromal and immune scores [17]. Subsequent researches on glioblastoma [18], colon cancer [19], and breast cancer [20] have been investigated with the ESTIMATE algorithm and shown good effectiveness of this algorithm. Nevertheless, research on ccRCC using the ESTIMATE algorithm has not been reported in detail.

In the present retrospective study, we applied the ESTIMATE algorithm for the analysis of gene expression profiles of ccRCC which were collected from The Cancer Genome Atlas (TCGA, https://cancergenome.nih.gov) to infer stromal and immune scores for the first time. The association of stromal and immune scores with clinicopathological parameter as well as clinical prognosis of ccRCC patients was also investigated.

## 2. Materials and Methods

### 2.1. Data Profile

The gene expression profiles of ccRCC were downloaded from TCGA (Apr 2019) and then were subjected to background correction and normalization with Perl 5.0 (http://www.perl.org/). Meanwhile, relevant clinical characteristics of cancer cases were also collected. Patients with follow-up time <30 days or lacking pathologic diagnosis would be removed.

### 2.2. ESTIMATE Algorithm

As described previously [17], with the ESTIMATE package in R (version 3.5.2, https://www.r-project.org), stromal and immune scores of each sample were calculated. The optimal cut-off values were evaluated with the online tool: Cutoff Finder (http://molpath.charite.de/cutoff/assign.jsp) [21]. Based on the cut-off values, patients were divided into low- and high-stromal/immune score groups. Group comparisons of stromal/immune scores between different clinical indexes were performed by the *t*-test with SPSS 20.0. *P* value < 0.05 was considered statistically significantly different.

### 2.3. Expression Analysis of Differentially Expressed Genes (DEGs)

The Bioconductor package, “edgeR” (http://www.bioconductor.org/packages/release/bioc/html/edgeR.html) was utilized to identify DEGs between low- and high-stromal/immune score groups. The overlapping DEGs would be used for further analysis.

### 2.4. Functional Enrichment Analysis and Protein-Protein Interaction (PPI) Network

All overlapping DEGs were utilized for Kyoto Encyclopedia of Genes and Genomes (KEGG) and Gene Ontology (GO) analysis with *P* value (adjusted *P* value) <0.05 as the threshold. In addition, the PPI network of overlapping DEGs would be obtained from STRING (https://string-db.org) [22] with confidence >0.7 as a cut-off criterion. Then, the data of the PPI network was reconstructed by Cytoscape (version 3.6, https://cytoscape.org), and the top five degree DEGs were selected as the most important targets. The relationship between hub genes and tumor-infiltrating immune cells (B cell, CD4 T cell, CD8 T cell, neutrophil, macrophage, and dendritic cell) was deciphered using TIMER (Tumor IMmune Estimation Resource, https://cistrome.shinyapps.io/timer/).

### 2.5. Survival Analysis

The Kaplan-Meier plots were conducted to illustrate survival difference between the low- and high-stromal/immune score groups with overall survival of ccRCC patients. Univariable and multivariable Cox regression model was used to determine independent prognostic factors. The *P* value < 0.05 was set as the cut-off value.

### 2.6. Expression Profile of Immunomodulators

In recent years, immune checkpoint inhibitors have been approved for the therapy of various cancers, including renal cell carcinoma [23]. In this study, several key immunomodulators (LAG-3, TIM-3, CTLA-4, IFN-*γ*, ICOS, ICAM-1, TIGIT, PD-1, PDL-1, NKG2A, and VISTA) were quantified in both normal kidney tissues and ccRCC tissues. The difference of expression of immunomodulators between normal and ccRCC samples as well as low- and high-stromal/immune score groups was compared by the *t*-test.

## 3. Results

### 3.1. Patient Characteristics

After removing cases with follow-up time <30 days, a total of 587 samples (72 normal kidney tissue samples and 515 ccRCC tissue samples) were collected from TCGA. The detailed demographic and baseline characteristics of 515 ccRCC patients are described in Table 1. All patients were diagnosed with ccRCC pathologically.

### 3.2. Evaluation of Immune Scores and Stromal Scores

**Figure 1:**
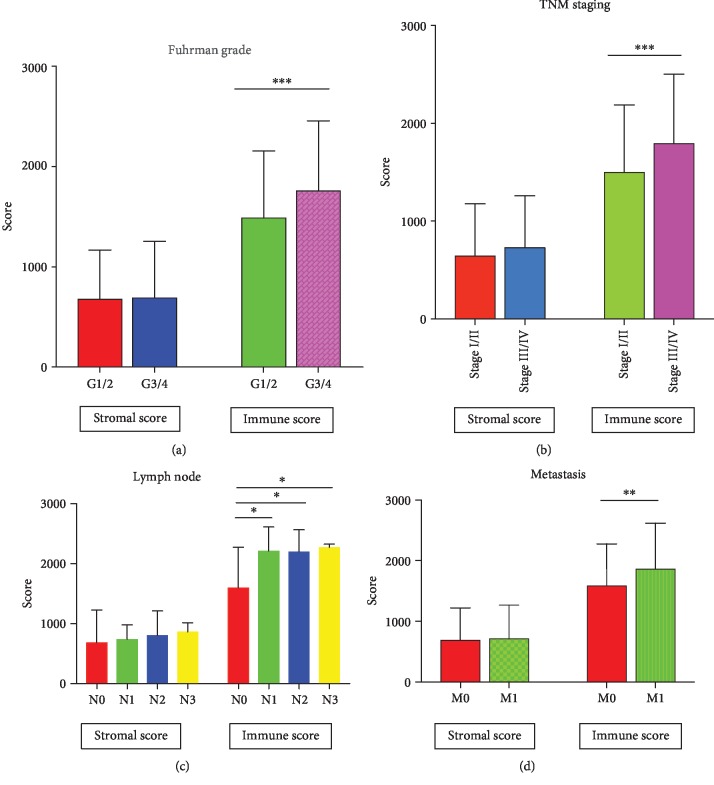
Distribution of stromal and immune scores. (a) Different Fuhrman grades and (b) TNM staging (c) with or without lymph node metastasis. N0, N1, N2, and N3 represent 0, 1, 2, and 3 lymph node metastases. (d) Distant metastasis.

Based on the cut-off values for stromal scores (841) or immune scores (1780), patients were assigned to low- and high-stromal/immune score groups. As shown in Figure 1(a), immune scores in Fuhrman grade 3/4 were significantly increased compared with those in Fuhrman grade 1/2 (*P* < 0.001). In addition, immune scores in stage iii/iv were also significantly increased (Figure 1(b), *P* < 0.001). However, for stromal scores, there was no statistical difference between different Fuhrman grades (*P* = 0.738) and TNM staging (*P* = 0.083). When compared with patients without lymph node metastasis, patients with 1, 2, and 3 lymph node metastases had higher immune scores (Figure 1(c), *P* = 0.012, *P* = 0.032, and *P* = 0.016). Similar results were observed in patients with distant metastasis (Figure 1(d), *P* = 0.001). Nevertheless, no difference of immune scores was observed between patients with or without lymph node metastasis (*P* = 0.529) and patients with or without distant metastasis (*P* = 0.685).

**Figure 2:**
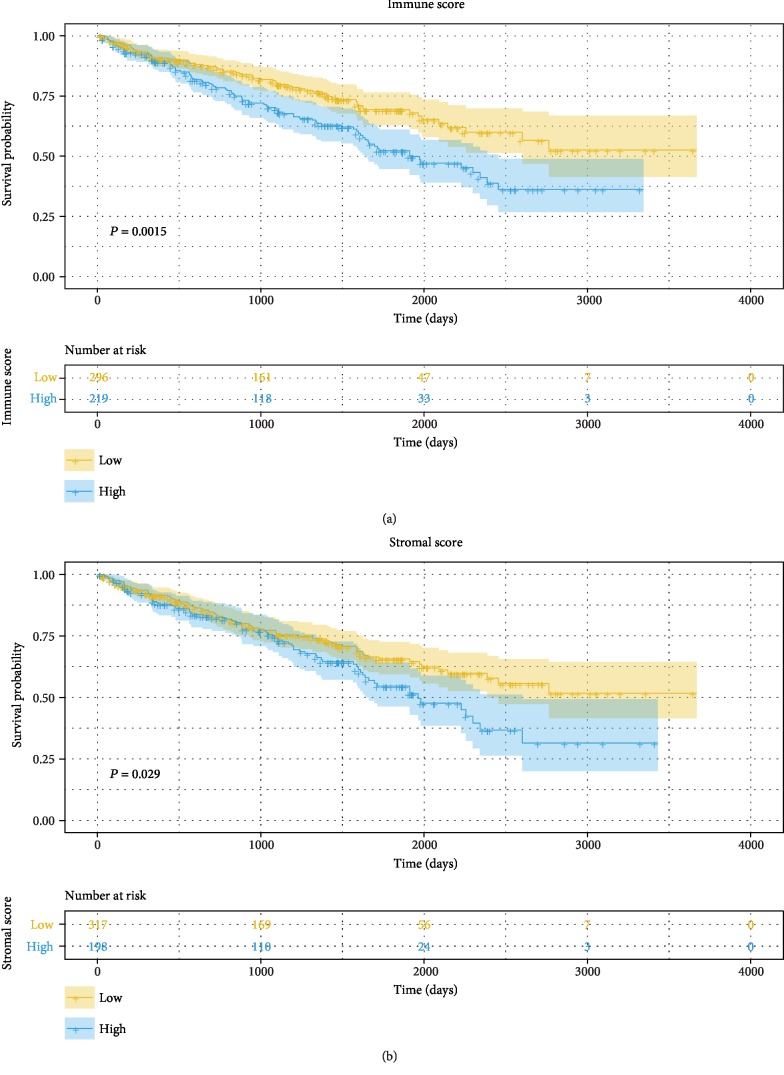
Prognostic values for overall survival: (a) immune scores and (b) stromal scores.

The associations of stromal/immune scores and corresponding overall survival were analyzed by the Kaplan-Meier plot and evaluated with the log-rank test. The Kaplan-Meier plot demonstrated that high-immune scores as well as stromal scores were negatively correlated with favorable outcome of ccRCC patients (Figure 2). In addition, 5-year survival rates of low- and high-immune score groups were 69.3% and 52.2%, respectively (HR = 1.659, 95% CI [1.204, 2.248]). And, for low- and high-stromal score groups, the rates were 65.7% and 54.6%, respectively (HR = 1.409, 95% CI [1.024, 1.938]). In addition, the result of multivariable Cox regression indicated that both stromal and immune scores were independent prognostic factors (Table 2).

### 3.3. Functional Enrichment Analysis and PPI Network

**Figure 3:**
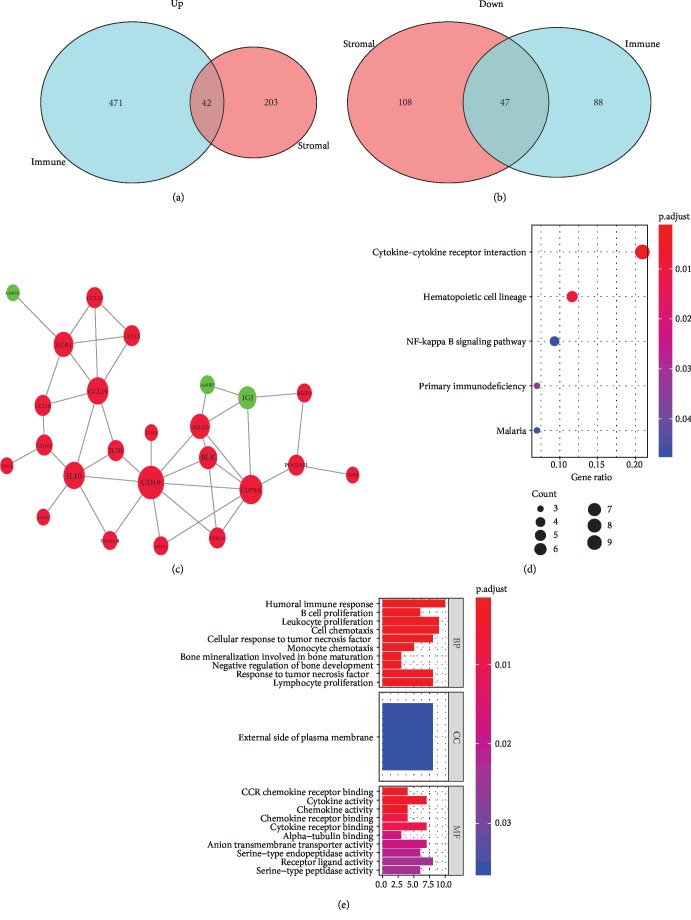
Analysis of DEGs. (a) 42 upregulated overlapping DEGs and (b) 47 downregulated overlapping DEGs. (c) Protein-protein interaction (PPI) network with confidence >0.7. Red and green nodes represent upregulated and downregulated genes, respectively. (d) The enriched pathways with *P* < 0.05. (e) The enriched GO terms with *P* < 0.05 and gene count >5.

A total of 89 overlapping DEGs were identified between low- and high-stromal/immune score groups, including 42 upregulated and 47 downregulated overlapping genes (Figures 3(a) and 3(b)). To better understand the role of DEGs, KEGG and GO analyses were carried out. KEGG analysis revealed 89 overlapping DEGs which were mainly involved in 5 pathways, such as cytokine-cytokine receptor interaction, NF kappa B signaling pathway, and primary immunodeficiency (Figure 3(d)). In addition, 84 GO terms (67 terms of biological process, 1 term of cellular component, and 16 terms of molecular function) were enriched (Figure 3(e), Supplemental [Supplementary-material supplementary-material-1]).

For exploring the interplay among 89 overlapping DEGs, a PPI network was built using the STRING tool with confidence >0.7 as the cut-off criterion. 24 nodes (21 upregulated and 3 downregulated DEGs) along with 39 edges consisted of the PPI network (Figure 3(c)). Moreover, in the PPI network, CD79a, CD19, CCL19, IL10, and XCR1 were the remarkable nodes as they had the most connections with other nodes. Furthermore, survival analysis revealed a significant correlation between the expression of IL10 (0.030) and XCR1 (*P* = 0.012) and prognosis of ccRCC (Supplemental [Supplementary-material supplementary-material-1]). In addition, the result showed that both IL10 and XCR1 were closely related to the infiltration of tumor immune cells (Supplemental [Supplementary-material supplementary-material-1]).

### 3.4. Expression Profile of Immunomodulators

**Figure 4:**
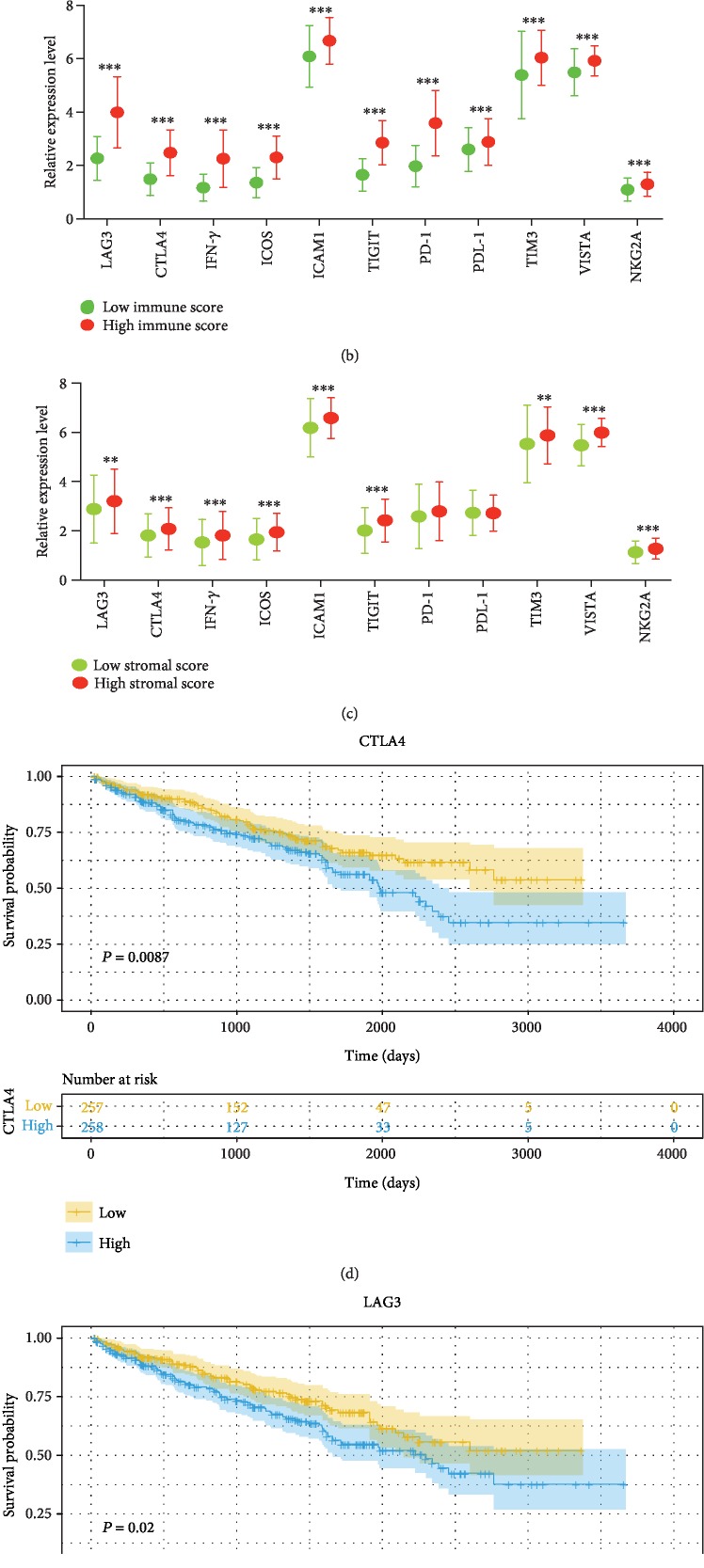
The distribution of immunomodulators (a) between normal samples and ccRCC samples, (b) low- and high-immune scores, and (c) low- and high-stromal scores. (d) Kaplan-Meier curves for overall survival of CTLA-4 and (e) LAG-3.

As shown in Figure 4(a), all of 11 immunomodulators (LAG-3, TIM-3, CTLA-4, IFN-*γ*, ICOS, ICAM-1, TIGIT, PD-1, PDL-1, NKG2A, and VISTA) were significantly increased in ccRCC samples compared with normal kidney samples. Moreover, in the high-stromal score group, the expression of 11 immunomodulators was all upregulated (Figure 4(b)). In the high-immune score group, similar phenomenon was observed except for PD-1 and PDL-1 (Figure 4(c)). Then, we further explored the prognostic value of 11 immunomodulators. Survival analysis suggested that patients with low expression of LAG3 or CTLA-4 had a longer overall survival than those with high expression of LAG3 (HR = 1.448, 95% CI [1.063, 1.972], Figure 4(d)) or CTLA-4 (HR = 1.513, 95% CI [1.11, 2.062], Figure 4(e)).

## 4. Discussion

ccRCC is one of the most prevalent kidney malignancies and accounts for approximately 3% of adult cancer [24, 25]. Previous studies have shown that the tumor microenvironment (TME) was highly related to the oncogenesis, progress, and prognosis of ccRCC [26]. TME is the place where the immune system and tumor interplay, reflecting the plasticity of both the tumor and immune system [27]. Tumor development is highly dependent on TME, and any alterations of the composition of TME may influence the evolution of malignancies [27]. Understanding the change may help the development of therapeutic strategies. Stromal cells and immune cells are the important components of TME, which play a critical role in the development of cancers [6]. The ESTIMATE algorithm, a tool based on a large scale of gene expression profile, has been used for the investigation of glioblastoma, colon cancer, and breast cancer and shown good precision and practicality [18–20]. However, it has not been applied for the research on ccRCC.

In the current study, we attempted to infer the level of infiltrating stromal and immune cells in ccRCC by calculating stromal and immune scores with the ESTIMATE algorithm. Survival curves according to Kaplan-Meier showed that low-immune scores as well as stromal scores predicted a favorable prognosis in ccRCC patients. In addition, evidence indicating a significant increase in immune scores was witnessed in patients in Fuhrman grade 3/4 and stage iii/iv, with lymph node metastasis as well as with distant metastasis, whereas for stromal scores, no differences were observed between different Fuhrman grades and TNM staging, with or without lymph node metastasis or distant metastasis.

For exploring the potential mechanism of the change of TME, we identified tumor microenvironment-related genes which would be further utilized for functional enrichment analysis and constructing the PPI network. A total of 89 overlapping differentially expressed genes (DEGs) (42 upregulated and 47 downregulated genes) were determined. KEGG analysis demonstrated that 5 pathways were enriched by 89 overlapping DEGs, including cytokine-cytokine receptor interaction, NF kappa B signaling pathway, and primary immunodeficiency. The NF kappa B signaling pathway is involved in immunity, inflammation, and cell survival [28]. Lua et al. found that the NF kappa B signaling pathway could affect the prognosis of RCC by decreasing the local inflammatory infiltrate and regulating TME [29]. Additionally, Morais et al. reported that inhibition of the NF kappa B signaling pathway attenuated the progression of RCC [30]. Meanwhile, the PPI network was constructed to predict the interaction relationship among 89 overlapping DEGs, and the top 5 degree genes (CD79a, CD19, CCL19, IL10, and XCR1) were extracted for further analysis. Among the top 5 degree genes, 2 DEGs (IL10 and XCR1) were highly relative to clinical outcome of ccRCC patients as well as the infiltration of tumor immune cells. IL10, a cytokine produced by monocytes and lymphocytes primarily, has pleiotropic effects on regulating immune response by stimulating B cells and inhibiting macrophages and helper T cells [31]. A previous study showed that the RCC patients with high expression of IL10 had a lower incidence of distant metastasis [32]. However, another research suggested that IL10-producing B cells were higher in advanced-stage RCC and could decrease the proportion of T cells in TME [33]. XCR1, the only receptor of the chemokine XCL1, is expressed in dendritic cells and has an implicated function in dendritic cell-mediated cytotoxic immune response [34]. Besides, the overexpression of XCR1 can promote the growth, migration, and invasion in breast cancer and non-small-cell lung cancer [35, 36]. All data suggested that IL10 and XCR1 may be potential key regulators for the TME of ccRCC and novel markers for the prognosis of ccRCC.

Recently, immune checkpoint inhibitors have evolved treatment strategies in oncology and have been approved for the therapy of various cancers including RCC [23]. In this study, we determined 11 immunomodulators that are involved in tumor escape mechanisms and found 9 immunomodulators (LAG-3, TIM-3, CTLA-4, IFN-*γ*, ICOS, ICAM-1, TIGIT, NKG2A, and VISTA) that were upregulated in both high-immune score group and high-stromal score group. In addition, PD-1 and PDL-1 were also increased in the high-stromal score group. Kaplan-Meier analysis demonstrated that longer overall survival among patients with low expression of LAG-3 and CTLA-4 was observed. CTLA-4, a homolog of CD28, is expressed by T cells and could inhibit the T cell immune response by diminishing costimulatory signal [37]. LAG-3 is expressed in activated CD4+ and CD8+ T cells and participates in helper T cell response [38]. Several researches have shown that LAG-3 expression was related to the metastasis and prognosis of various cancers such as breast cancer, lung cancer, and ovarian cancer [39–41].

There are a few limitations to be addressed in this study. Firstly, due to the fact that all patients were gathered from TCGA database, the potential of selection bias could not be excluded and it was not possible to collect all information of patients, such as the organ distributions with metastasis and information on whether to use anti-inflammatory drugs or not. Secondly, there was no experimental research conducted to examine the functions of IL10 and XCR1 in ccRCC. Thus, further investigation both in vitro and in vivo is demanded to testify the discovery of this research.

In summary, we applied the ESTIMATE algorithm to calculate stromal and immune scores which were highly associated with the clinical outcome of ccRCC. In addition, we identified 89 microenvironment-related genes, and data from the PPI network and survival analysis revealed that IL10 and XCR1 may the potential key regulators for the TME of ccRCC and could be useful for outlining the prognosis of ccRCC patients. However, more experimental research both in vitro and in vivo is needed to examine the finding of this research.

## Figures and Tables

**Table 1:** The baseline characteristics of all patients.

Parameter	Subtype	No.	Percent (%)
Age (years)	<65	250	48.5
	≥65	265	51.6
Gender	Male	341	66.2
	Female	174	33.8
TNM staging	Stage i/ii	315	61.2
	Stage iii/iv	200	38.8
Fuhrman grade	G 1/2	235	45.6
	G 3/4	280	54.4
Immune score	Low	296	57.5
	High	219	42.5
Stromal score	Low	317	61.6
	High	198	38.4
Survival status	Alive	354	68.7
	Dead	161	31.3
Total		515	100

**Table 2:** Results of univariate and multivariable Cox regression analysis.

Characteristics	Univariate analysis			Multivariate analysis		
	HR	95% CI	*P*	HR	95% CI	*P*
Age (<65/≥65)	1.690	(1.232, 2.318)	0.001	1.512	(1.096, 2.087)	0.012
Sex (female/male)	0.937	(0.680, 1.292)	0.691	0.921	(0.665, 1.275)	0.619
Fuhrman grade (1 and2/3 and 4)	2.742	(1.923, 3.911)	<0.001	1.771	(1.218, 2.574)	0.003
Stage (I and ii/iii and iv)	4.361	(3.162, 6.085)	<0.001	3.410	(2.394, 4.857)	<0.001
Stromal scores (low/high)	1.411	(1.134, 1.925)	0.030	1.236	(1.110, 1.551)	0.045
Immune scores (low/high)	1.647	(1.207, 2.248)	0.002	1.321	(1.197, 1.671)	0.029

HR: hazard ratio; 95% CI: 95% confidence interval.

## Data Availability

The data used to support the findings of this study are included within the article.

## Supplementary Materials

Supplementary Materials Supplemental Table 1 Gene Ontology analysis of 89 overlapping DEGs. Supplemental Figure 1: prognostic values for overall survival. (A) IL10. (B) XCR1. Supplemental Figure 2: the relationship between hub genes and tumor-infiltrating immune cells (B cell, CD4 T cell, CD8 T cell, neutrophil, macrophage, and dendritic cell).
