# Supplementary material for: Data Mining of Prognostic Microenvironment-Related Genes in Clear Cell Renal Cell Carcinoma: A Study with TCGA Database
Source: Dis Markers. 2019 Oct 30;2019:8901649. doi: 10.1155/2019/8901649 (PMC6875323; doi:10.1155/2019/8901649)
Supplement: Supplementary Materials — Supplemental Table 1 Gene Ontology analysis of 89 overlapping DEGs. Supplemental Figure 1: prognostic values for overall survival. (A) IL10. (B) XCR1. Supplemental Figure 2: the relationship between hub genes and tumor-infiltrating immune cells (B cell, CD4 T cell, CD8 T cell, neutrophil, macrophage, and dendritic cell). [file 8901649.f1.pdf]

Supplemental Table 1. Gene Ontology analysis of 89 overlapping DEGs

GO	terms	GeneRatio	Count	p.adjust
BP	humoral immune response	349/18493	10	0.002467333
BP	B cell proliferation	91/18493	6	0.002467333
BP	leukocyte proliferation	283/18493	9	0.002467333
BP	cell chemotaxis	295/18493	9	0.002591478
BP	cellular response to tumor necrosis factor	235/18493	8	0.00270804
BP	monocyte chemotaxis	64/18493	5	0.00270804
BP	bone mineralization involved in bone maturation	11/18493	3	0.00298566
BP	negative regulation of bone development	11/18493	3	0.00298566
BP	response to tumor necrosis factor	257/18493	8	0.003407588
BP	lymphocyte proliferation	264/18493	8	0.003570846
BP	mononuclear cell proliferation	266/18493	8	0.003570846
BP	mononuclear cell migration	85/18493	5	0.005421627
BP	chemokine-mediated signaling pathway	87/18493	5	0.005599111
BP	leukocyte chemotaxis	217/18493	7	0.00606826
BP	response to chemokine	95/18493	5	0.00641872
BP	cellular response to chemokine	95/18493	5	0.00641872
BP	glucocorticoid biosynthetic process	18/18493	3	0.00641872
BP	ossification involved in bone maturation	18/18493	3	0.00641872
BP	bone maturation	20/18493	3	0.008441835
BP	leukocyte apoptotic process	107/18493	5	0.00977991
BP	negative regulation of immune system process	437/18493	9	0.010704336
BP	animal organ maturation	24/18493	3	0.012225896
BP	regulation of bone development	24/18493	3	0.012225896
BP	lymphocyte chemotaxis	63/18493	4	0.012741324
BP	glucocorticoid metabolic process	25/18493	3	0.012741324
BP	regulation of lymphocyte activation	472/18493	9	0.015384142

BP	negative regulation of immune response	131/18493	5	0.018354427
BP	cell-cell recognition	72/18493	4	0.018354427
BP	B cell activation	303/18493	7	0.021829088
BP	regulation of leukocyte proliferation	216/18493	6	0.021829088
BP	regulation of T cell activation	305/18493	7	0.021829088
BP	animal organ regeneration	78/18493	4	0.021829088
BP	regulation of adaptive immune response based on somatic recombination of immune receptors built from immunoglobulin superfamily domains	144/18493	5	0.023641456
BP	hormone biosynthetic process	84/18493	4	0.027244111
BP	regulation of leukocyte apoptotic process	87/18493	4	0.030229446
BP	regulation of adaptive immune response	157/18493	5	0.032129966
BP	receptor-mediated endocytosis	346/18493	7	0.036395478
BP	positive regulation of cell-cell adhesion	251/18493	6	0.036395478
BP	establishment of T cell polarity	10/18493	2	0.036395478
BP	dendritic cell antigen processing and presentation	10/18493	2	0.036395478
BP	dendritic cell apoptotic process	10/18493	2	0.036395478
BP	regulation of dendritic cell apoptotic process	10/18493	2	0.036395478
BP	positive regulation of protein kinase B signaling	168/18493	5	0.036508724
BP	neutrophil chemotaxis	101/18493	4	0.040499856
BP	establishment of lymphocyte polarity	11/18493	2	0.040499856
BP	creatine metabolic process	11/18493	2	0.040499856
BP	antibacterial humoral response	47/18493	3	0.043691536
BP	leukocyte migration	478/18493	8	0.043691536
BP	lymphocyte migration	105/18493	4	0.043691536
BP	developmental maturation	271/18493	6	0.043933972
BP	positive regulation of leukocyte activation	372/18493	7	0.043933972
BP	regulation of leukocyte chemotaxis	109/18493	4	0.046862642
BP	immunological synapse formation	13/18493	2	0.046862642

BP	negative regulation of immune effector process	111/18493	4	0.046862642
BP	positive regulation of receptor-mediated endocytosis	51/18493	3	0.046862642
BP	negative regulation of leukocyte apoptotic process	51/18493	3	0.046862642
BP	positive regulation of cell activation	384/18493	7	0.046862642
BP	neutrophil migration	112/18493	4	0.046862642
BP	steroid biosynthetic process	193/18493	5	0.04847223
BP	regulation of ossification	193/18493	5	0.04847223
BP	protein activation cascade	194/18493	5	0.048773173
BP	complement activation, alternative pathway	14/18493	2	0.049293986
BP	T cell costimulation	54/18493	3	0.049647673
BP	antimicrobial humoral response	118/18493	4	0.049647673
BP	lymphocyte costimulation	55/18493	3	0.049647673
BP	regulation of leukocyte cell-cell adhesion	293/18493	6	0.049647673
BP	myeloid leukocyte migration	199/18493	5	0.049647673
CC	external side of plasma membrane	359/19659	8	0.036152847
MF	CCR chemokine receptor binding	43/17632	4	0.0048119
MF	cytokine activity	219/17632	7	0.0048119
MF	chemokine activity	49/17632	4	0.0048119
MF	chemokine receptor binding	66/17632	4	0.009639388
MF	cytokine receptor binding	281/17632	7	0.009639388
MF	alpha-tubulin binding	34/17632	3	0.01772548
MF	anion transmembrane transporter activity	330/17632	7	0.01822859
MF	serine-type endopeptidase activity	250/17632	6	0.021776712
MF	receptor ligand activity	478/17632	8	0.0236094
MF	serine-type peptidase activity	273/17632	6	0.0236094
MF	serine hydrolase activity	277/17632	6	0.0236094
MF	voltage-gated chloride channel activity	12/17632	2	0.0236094
MF	peroxisome proliferator activated receptor binding	12/17632	2	0.0236094
MF	phosphotransferase activity, nitrogenous group as acceptor	13/17632	2	0.025838687

MF	growth factor receptor binding	132/17632	4	0.042231687
MF	voltage-gated anion channel activity	18/17632	2	0.043750023

GO; Gene Ontology, DEGs; differentially expressed genes, BP; Biological Process, CC; Cellular Component, MF; Molecular Function

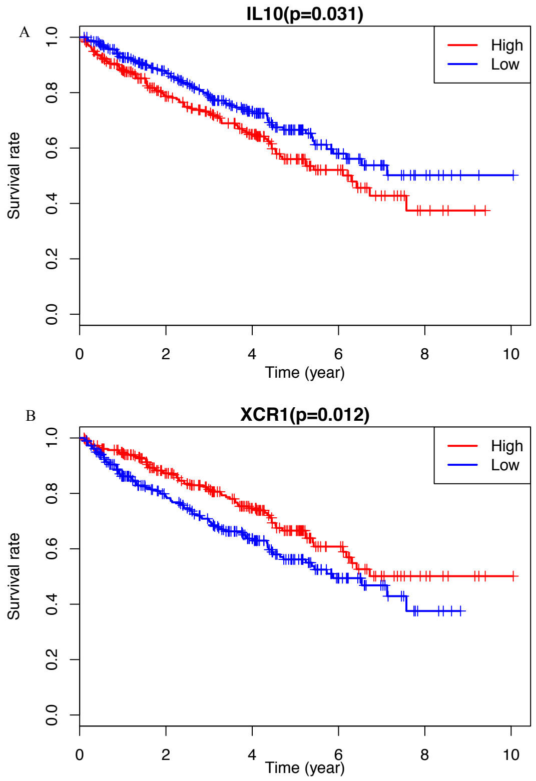

Supplemental Figure 1. Prognostic values for overall survival. (A) IL10. (B) XCR1.

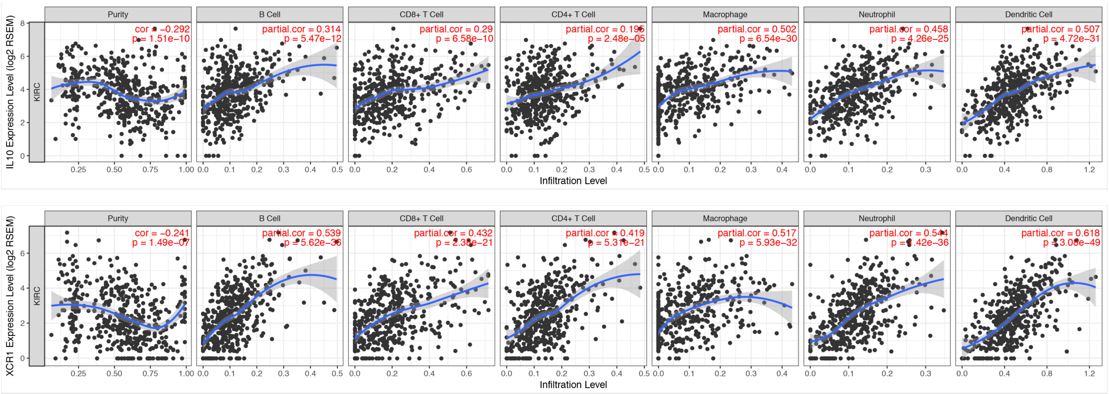

Supplemental Figure 2. The relationship between hub genes and tumor infiltrating immune cells (B cell, CD4 T cell, CD8 T cell, neutrphil, macrophage and dendritic cell).
